# Calcium Transients at ER Subdomains Initiate Autophagosome Formation: A Single Spark Can Start a Prairie Fire

**DOI:** 10.1093/function/zqad004

**Published:** 2023-01-27

**Authors:** Shuang Peng

**Affiliations:** School of Sport and Health Sciences, Guangzhou Sport University, Guangzhou 510500, China; Key Laboratory of Sports Technique, Tactics and Physical Function of General Administration of Sport of China, Scientific Research Center, Guangzhou Sport University, Guangzhou 510500, China

**Keywords:** Ca^2+^ signaling, autophagosome, endoplasmic reticulum, FIP200 puncta, EI24

Autophagy is an evolutionarily conserved and tightly regulated lysosome-mediated intracellular bulk degradation pathway by which intracellular macromolecules are sequestered in autophagosomes and delivered to lysosomes for degradation and recycling. Identification of autophagy-related (ATG) genes in yeast has promoted the understanding of the molecular mechanism of autophagosome formation.^1^ The proteins encoded by these genes play a crucial role at different steps of autophagosome formation. For example, Atg17/Atg13/Atg1 complexes form condensates and localize on the vacuole membrane, thereby recruiting downstream autophagy proteins to promote the formation of the isolation membrane on the vacuole.^2^ Autophagosome biogenesis involves nucleation, expansion, and closure of the isolation membrane.

Calcium (Ca^2+^) is well known as an essential second messenger in eukaryotic cells.^3^ Ca^2+^ levels are distinct in different subcellular compartments and are built up by Ca^2+^ channels and pumps located in the plasma membrane and organelles. Due to the resulting highly localized gradients, cytoplasmic Ca^2+^ signals display spatiotemporal heterogeneity in the form of sparks, transients, oscillations, and waves. It is well known that rapid dynamic changes of the local Ca^2+^ concentration ([Ca^2+^]) in the cytoplasm and organelles are capable of initiating a variety of physiological signals in the cell. Mounting evidence has shown that cytosolic and endoplasmic reticulum (ER) Ca^2+^ transients are the core signals in the regulation of autophagy activity. One of the important differences in autophagy between metazoans and yeast is the location of autophagosome formation. In yeast, autophagosomes fuse with the tonoplast and are formed at a single site,^4^ whereas in mammalian cells, autophagosomes are initially formed simultaneously at multiple sites on the ER.^5^ The assembly and activation of the FIP200/ATG13/ULK1 complex at these sites is a key step in initiating the initial autophagic membrane formation.^6^ However, it is still unclear how autophagy signaling leads to activation of the ULK1 complex at specific sites on the ER. Furthermore, previous studies have shown that changes of [Ca^2+^]_c_ and [Ca^2+^]_ER_ could regulate autophagy by affecting the activity of proteins in the autophagic machinery.**^7^** Nevertheless, the spatiotemporal patterning of cellular Ca^2+^ signals and the molecular mechanisms involved in the regulation of the autophagosome during the process of autophagy are completely unknown. Therefore, identification of how Ca^2+^ signals regulate autophagosome formation on the ER is an unresolved scientific question in the field of autophagy.

Recently, Zhang and colleagues^8^ revealed how Ca^2+^ signals trigger the assembly of the autophagosome-initiating FIP200 complex on the ER. They showed that upon autophagy induction, Ca^2+^ transients on the ER surface are key signals determining autophagosome formation. They also demonstrated that Ca^2+^ transients on the ER surface trigger liquid–liquid phase separation of the FIP200 complex, and that subsequently the formed FIP200 puncta aggregate to bind to the ER membrane proteins VAPA/B (VAPs) and Atlastin 2/3 (ATLs). These proteins are localized at the ER and become autophagosome initiation sites (Figure 1).

**Figure 1.**
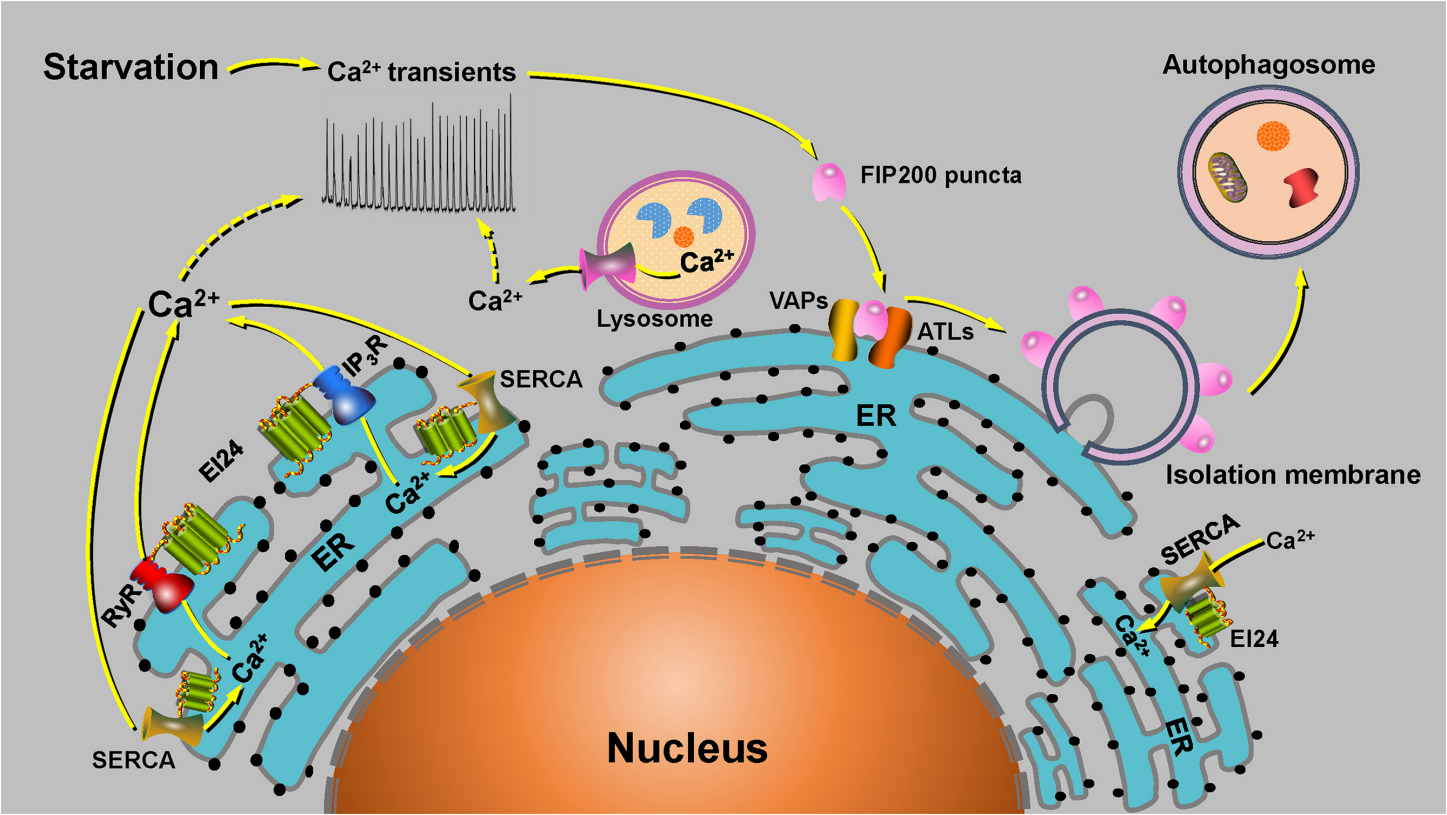
Schematic diagram illustrating that the metazoan-specific ER transmembrane autophagy protein EI24 interacts with Ca^2+^ channels and pumps in combination with lysosomal Ca^2+^ release to generate Ca^2+^ transients on the ER outer surface to induce liquid-like FIP200 puncta for autophagosome formation. See text for further explanation.

Zheng et al.^8^ found that BAPTA-AM, a fast calcium chelator, was able to inhibit the formation of FIP200 condensates on the ER, whereas this process could not be blocked by the slow calcium chelator EGTA-AM. This suggests that fast and local Ca^2+^ signals, rather than steady-state changes in Ca^2+^ concentration, may be involved in the initiation of autophagy. In order to assess local Ca^2+^ perturbations in a submicrometer domain on the ER, they constructed a new biosensor, CYB5-GCaMP6f, to detect autophagy-induced Ca^2+^ changes in a narrow spatial domain on the outer surface of the ER. Analysis by multimodal structured illumination microscopy (multi-SIM) revealed that Ca^2+^ transients or oscillations, which could be blocked by BAPTA-AM, occurred on the ER surface under starvation or Torin1 treatment. Further experiments using pharmacological inhibition of Ca^2+^ release channels, or knockdown strategies, showed that FIP200 condensates on the ER were significantly reduced.^8^ This indicates that Ca^2+^ transients on the ER surface are essential for autophagosome initiation. In contrast, Ca^2+^ release channel activator treatment increased the frequency, amplitude, and duration of local/global Ca^2+^ transients and oscillations on the ER. However, autophagic flux activity was inhibited in this scenario, suggesting that persistent activation of calcium release channels suppress the formation of functional autophagic structures. This conclusion was also supported by transmission electron microscopy studies demonstrating that ER-associated small unacidified autophagosomes exist in Ca^2+^ release channel activator-treated cells.

Zhang and colleagues previously identified a series of autophagy genes unique to multicellular organisms such as *EPG-4/EI24*.^9^ In their recent study,^8^ the pattern of Ca^2+^ signals on the ER surface in *EI24* knockout cells was similar to what was observed in the cells treated with a calcium channel activator. Moreover, *EI24* knockout caused autophagy defects, which could be attenuated by the application of Ca^2+^ release channel inhibitors or knockdown. Most importantly, Zheng et al.^8^ also discovered that EI24 interacted with the Ca^2+^ release channels and pumps on the ER membrane, causing clustering of a subtype of inositol 1,4,5-trisphosphate receptor (IP_3_R)-IP_3_R_3_ to modulate ER Ca^2+^ transients. Additionally, they found that Ca^2+^ release from lysosomes was also involved in the formation of FIP200 puncta on the ER. Ca^2+^ transients on the ER surface were further shown to promote the assembly of the ULK1 complex by triggering the formation of ATG13-dependent FIP200 liquid–liquid phase separation, in which VAP and ATL2/3 proteins play an important role. Finally, ATG9 vesicles were found to regulate this phase separation and the spatial organization of FIP200 puncta on the ER. This indicates an important functional role of ATG9–ULK1 interaction in autophagosome formation.

Ca^2+^ release has now been shown to significantly determine the ER Ca^2+^ transients that trigger the formation of FIP200 puncta upon autophagy induction, but it will be important to extend the work of Zheng et al. by exploring the possible role of other Ca^2+^ channels, such as Ca^2+^ entry channels, which may modulate the ER Ca^2+^ transients. It would also be interesting to investigate the potential mechanism of the generation of ER Ca^2+^ transients stimulated by starvation and explore how these signals trigger formation of FIP200 puncta.

## Data Availability

There are no data presented in this perspective.
